# Two Cases of Internal Injury from a Fall

**Published:** 1859-01

**Authors:** B. F. Schneck

**Affiliations:** Lebanon, Penn.


ARTICLE VIII.

### TWO CASES OF INTERNAL INJURY FROM A FALL; THEIR OPPOSITE AND UNEXPECTED TERMINATIONS.

BY B. F. SCHNECK, M. D., LEBANON, PENN.

CASE 1.—April 7th, 1858, was called to the wife of Wm. Hoffman, who was said to have fallen in the following singular way: engaged in lifting a heavy iron kettle from the fire while washing, she slipped, fell, and struck with her left side the edge of a cedar bucket. The fall was thus only from the perpendicular to the horizontal. She was taken up insensible. I found her recovered, it is true, but alarmingly ill. There was no pulse at the wrist; no throbbing of the carotids; a mere flutter audible at the heart; the entire body as cold and white as that of a corpse; and the lips, even, perfectly blanched. Indeed, the shock to the system was so great that the woman threatened to die every moment. This prostration may have been owing to the solar plexus of nerves having been greatly affected by the blow. She complained also of a stitch in the left side, which was much benefited by a tight bandage. Brandy was freely used; sinapisms, hot bricks, etc., were applied to the extremities; and yet for two days and a night the woman lay in the same collapsed state, though perfectly conscious and rational. On the third day she vomited, and complained of severe pain in the abdomen, which began to swell enormously, and a real peritonitis supervened. On the fifth day the tongue became dry, a profuse watery diarrhœa set in, and the symptoms of enteritis were clearly declared.

During all this time the treatment was fomentations, opium and calomel, brandy and nourishment; the stimulants, though contra-indicated by the inflammatory action, being nevertheless required by the extreme collapse which was ever present.

*April 15th.*—Pulse very small; action of the heart feeble; skin cool, and surface pale; considerable dysuria and tenesmus; abdomen softer, reduced in size, and less painful. Gave pl. acet. 2 grs., opium 1 gr., cret. preparat. 3 grs., after every evacuation; and quinia sul. four times a day, with brandy and beef-tea freely.

*April 20th, 25th.*—Slowly convalescing; able to sit up.

CASE 2.—On the evening of April 10th, 1858, John Kindig, aged about 40 years, fell from a tree which he was pruning, a height of fifteen feet. I saw him in probably half an hour afterwards, and found him as follows: Fracture of the left radius close to the hand; a cut in the scalp, about two inches long, a little above the left ear, and running parallel with the temporal ridge. His general aspect was very favorable; his warmth and color unaffected; no pallor, as in the first case, indicating any shock to the system; the pulse slow and regular, and no difficulty about the heart perceptible to the ear. A deep inspiration caused some pain across the chest, but more a feeling of constriction. There was no cough. He complained of some stiffness of his neck, and moved his legs freely. Save the injuries above stated, the entire organism appeared, after the most careful scrutiny, to have almost miraculously escaped; and the investigation was certainly thorough, for the recollection of the woman's fall a few days before was too fresh in my mind to have permitted me to be at all careless. There was no concussion nor compression of the brain here; no depression of the bone, as his rational conversation indicated. Until I had dressed the fracture and the flesh wound there was consumed at least an hour, or more; and during all this time I could distinguish no other lesion. I was informed, however, that at 4 o'clock the next morning he had some spasms or nervous twitchings, as they were described to me, and immediately died. I was also told that about midnight he had a chill, after which a slight reaction came on; but as he felt well, neither he nor his attendants had any presentiment of death, until it quietly and quickly came upon him.

One thing is remarkable: he felt no pain while I was adjusting the fracture, although the displacement was such as must have caused not a little suffering during the necessary manipulations.

What was here the cause of death? No post-mortem examination having been allowed, we must only conjecture. It was not concussion or compression of the brain; not dislocation of the vertebræ; but it may have been rupture of the liver, of the spleen, of a great bloodvessel, or of the stomach.

On the following morning, when I was shocked to find him dead, I felt his abdomen, and found it very much tumefied, and presenting a hard and *doughy* sensation to the touch.

